# The cost-effectiveness of specialized nursing interventions for people with Parkinson’s disease: the NICE-PD study protocol for a randomized controlled clinical trial

**DOI:** 10.1186/s13063-019-3926-y

**Published:** 2020-01-15

**Authors:** Danique L. M. Radder, Herma H. Lennaerts, Hester Vermeulen, Thies van Asseldonk, Cathérine C. S. Delnooz, Rob H. Hagen, Marten Munneke, Bastiaan R. Bloem, Nienke M. de Vries

**Affiliations:** 1 Department of Neurology, Donders Institute for Brain, Cognition and Behavior, Radboud University Medical Center, PO Box 9101 (947), 6500 HB Nijmegen, The Netherlands; 2 Department of Neurology, Department of Anesthesiology, Pain and Palliative Care, Radboud University Medical Center, Nijmegen, The Netherlands; 3 Radboud Institute for Health Sciences, IQ Healthcare, Radboud University Medical Center, Nijmegen, The Netherlands; 4 Department of Neurology, Elisabeth-TweeSteden Center, Tilburg, The Netherlands; 5 Department of Neurology, Máxima Medical Center, Veldhoven, The Netherlands; 6 Patient Expert at the Dutch Parkinson Association, Bunnik, The Netherlands

**Keywords:** Parkinson’s disease, Parkinson’s disease nurse specialist, Nursing, Quality of life, Cost-effectiveness, Multidisciplinary care

## Abstract

**Background:**

Current guidelines recommend that every person with Parkinson’s disease (PD) should have access to Parkinson’s disease nurse specialist (PDNS) care. However, there is little scientific evidence of the cost-effectiveness of PDNS care. This hampers wider implementation, creates unequal access to care, and possibly leads to avoidable disability and costs. Therefore, we aim to study the (cost-)effectiveness of specialized nursing care provided by a PDNS compared with usual care (without PDNS) for people with PD in all disease stages. To gain more insight into the deployed interventions and their effects, a preplanned subgroup analysis will be performed on the basis of disease duration (diagnosis < 5, 5–10, or > 10 years ago).

**Methods:**

We will perform an 18-month, single-blind, randomized controlled clinical trial in eight community hospitals in the Netherlands. A total of 240 people with PD who have not been treated by a PDNS over the past 2 years will be included, independent of disease severity or duration. In each hospital, 30 patients will randomly be allocated in a 1:1 ratio to receive either care by a PDNS (who works according to a recent guideline on PDNS care) or usual care. We will use two co-primary outcomes: quality of life (measured with the Parkinson’s Disease Questionnaire-39) and motor symptoms (measured with the Movement Disorders Society-sponsored revision of the Unified Parkinson’s Disease Rating Scale part III). Secondary outcomes include nonmotor symptoms, health-related quality of life, experienced quality of care, self-management, medication adherence, caregiver burden, and coping skills. Data will be collected after 12 months and 18 months by a blinded researcher. A healthcare utilization and productivity loss questionnaire will be completed every 3 months.

**Discussion:**

The results of this trial will have an immediate impact on the current care of people with PD. We hypothesize that by offering more patients access to PDNS care, quality of life will increase. We also expect healthcare costs to remain equal because increases in direct medical costs (funding additional nurses) will be offset by a reduced number of consultations with the general practitioner and neurologist. If these outcomes are reached, wide implementation of PDNS care will be warranted.

**Trial registration:**

ClinicalTrials.gov, NCT03830190. Registered February 5, 2019 (retrospectively registered).

## Background

Parkinson’s disease (PD) is a complex, progressive neurodegenerative disorder. Despite optimal medical management, such as with dopaminergic medication or deep brain stimulation (DBS) [1], most persons with PD experience progressively increasing disabilities that influence the quality of life of both patients and their caregivers [2, 3]. PD is characterized by a wide range of motor and nonmotor symptoms, including bradykinesia, tremor, rigidity, gait disturbances, psychiatric symptoms, and autonomic and cognitive dysfunction [4]. Many of these symptoms (i.e., freezing of gait, postural instability, and a wide range of nonmotor symptoms) are poorly controlled by medication [5]. The complexity of the disease in combination with limited treatment options creates tremendous challenges for the management of PD [6] and in coping with the disease for patients and their caregivers [7, 8].

In primary care, improved collaboration between doctors and nurses may lead to more integrated and consequently better-quality care. Indeed, there is increasing evidence that care delivered by trained nurses may generate similar or possibly better health outcomes for a wide range of disorders [9]. For the specific situation of PD, the Parkinson’s disease nurse specialist (PDNS) might fulfill a pivotal role in the multidisciplinary team. The PDNS was introduced in 1989 in the United Kingdom to bridge the gap between medical management and the unique personal needs of patients [10]. To obtain greater uniformity in care delivery and to facilitate the efficacy of nursing care in PD, the Dutch guideline “Nursing care in Parkinson’s disease” was published in 2015 [11]. The main roles of the PDNS are clearly described in this guideline, including (1) providing information, education, and instruction; (2) supporting the patient and caregiver in the promotion of self-management; (3) supporting psychosocial care questions; (4) prevention; (5) specialized diagnostic strategies and therapeutic nursing interventions; and (6) multidisciplinary collaboration.

Based on expert opinion from healthcare professionals, the Dutch guideline recommends that every person with PD could benefit from PDNS care, including those in early-stage disease, where information delivery, education about medication compliance, and support in self-management are crucial. So far, only three studies have evaluated PDNS care, with inconsistent results. Overall, the findings suggested that PDNS care may improve patient well-being, physical functioning, and general health status and reduce anxiety and depression [12–14], but definite conclusions cannot be drawn. Moreover, there is little evidence to show that quality of life actually improves with PDNS care. Finally, to date, no studies have evaluated the cost-effectiveness of PDNS care [9].

Presumably because the scientific evidence is inconclusive, many centers currently lack the nursing capacity and financial resources to offer PDNS care to all patients. This situation creates an undesirable inequality in access to care and presumably leads to avoidable disability and costs (e.g., from early admissions to nursing homes or crisis admissions to the hospital). Therefore, we aim to study the cost-effectiveness of specialized nursing care provided by a PDNS as compared with no PDNS care for people with PD. We hypothesize that offering PDNS care will lead to higher quality of life [15, 16]. We also expect healthcare costs to remain equal, because any increases in direct medical costs (to fund the extra nurse staffing) will be offset by a reduced number of (telephone) consultations with the primary care physician and neurologist. When this hypothesis is confirmed, wide implementation of PDNS care for patients with PD in all disease stages will be warranted. Conversely, negative findings would necessitate a critical reappraisal of the role of PDNS care as it is defined and delivered in its current form.

## Methods

### Study design

**Fig. 1:**
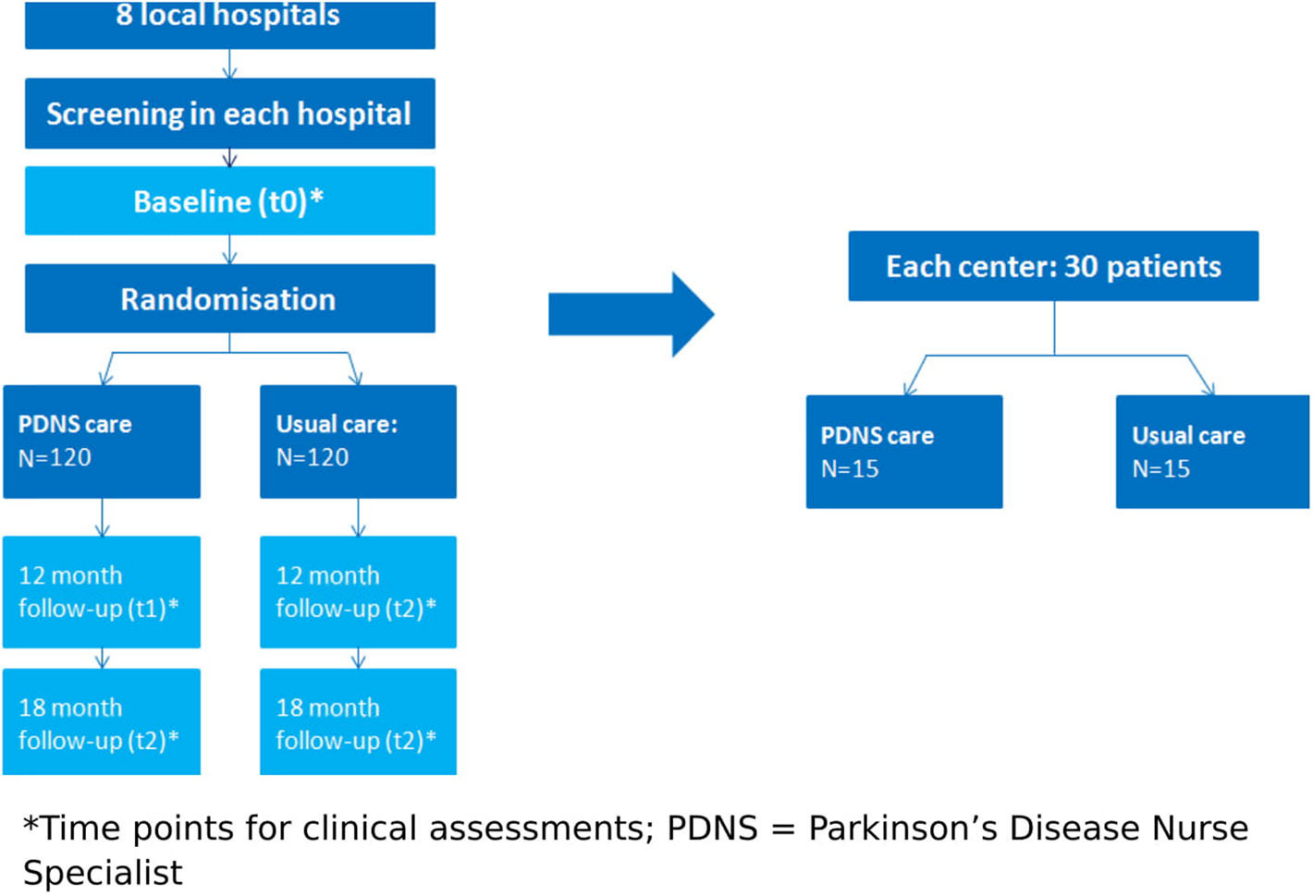
Summary of the study design. *Time points for clinical assessments. *PDNS* Parkinson’s disease nurse specialist

**Fig. 2:**
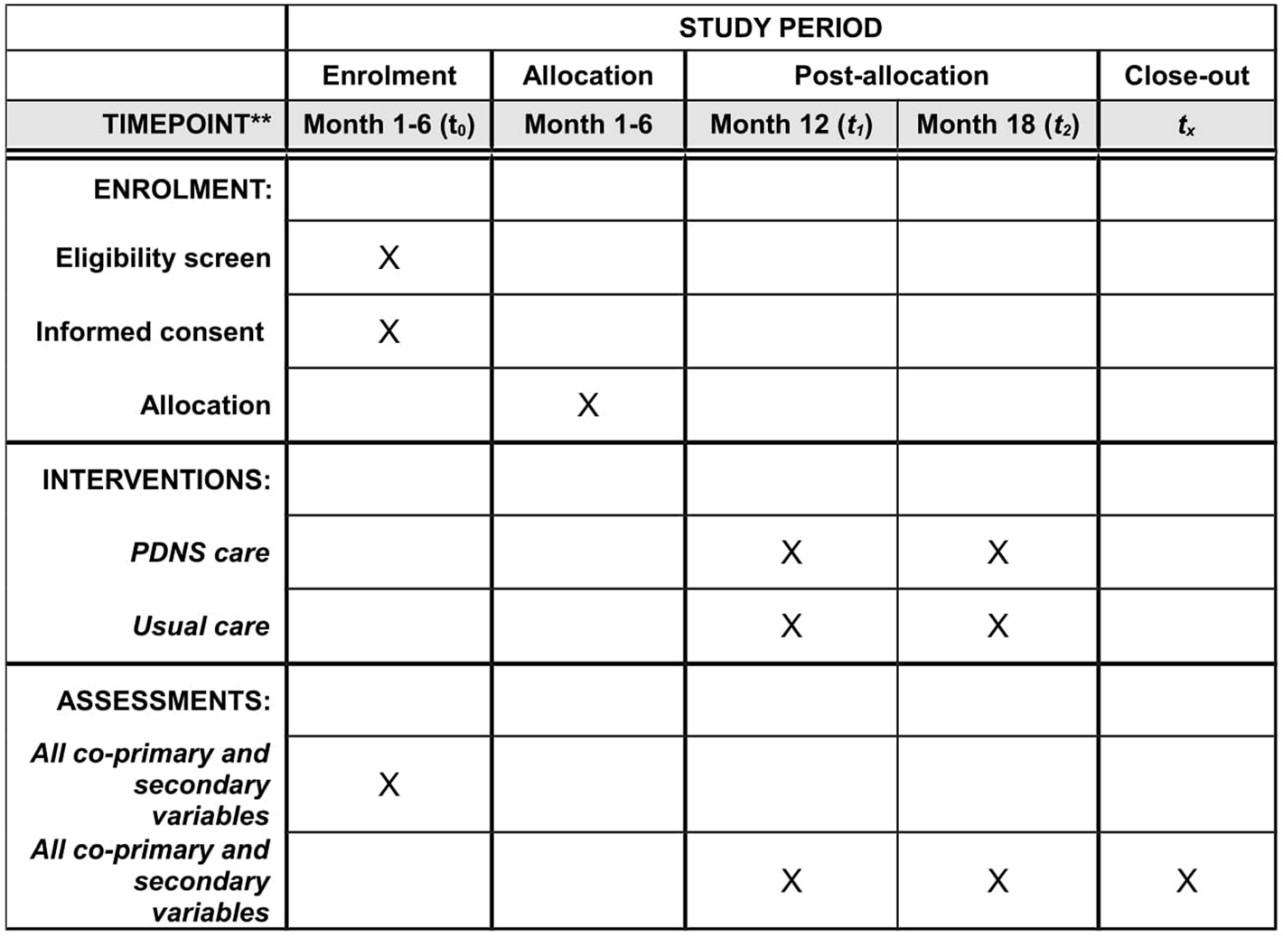
Example template of recommended content for the schedule of enrollment, interventions, and assessments

The Cost-effectiveness of Nursing Interventions for Patients With PD (NICE-PD) study protocol is reported here according to the Standard Protocol Items*:* Recommendations for Interventional Trials (SPIRIT) 2013 Statement [17]. Additional file 1 details the NICE-PD SPIRIT checklist. The study is an 18-month, single-blind, randomized controlled clinical trial that will be performed in eight community hospitals in the Netherlands. The participating centers are listed in Table 1. A total of 240 people with PD will be included (120 in each group), equally distributed over the participating hospitals. We have selected hospitals where, due to lack of sufficient PDNS staff, only a proportion of patients with PD currently have access to PDNS care. This provides us with a unique opportunity to identify patients who currently have no access to PDNS care and to randomize them within hospitals (at the patient level) to PDNS care or to no nursing intervention. We summarize the study design in Fig. 1. The enrollment and assessments during the study period are shown in Fig. 2.

**Table 1:** Participating community hospitals in the Netherlands

Center	Location
BovenIJ Hospital	Amsterdam
Treant Care Group, location Scheeper	Emmen
Elisabeth-TweeSteden Hospital	Tilburg
St. Jans Gasthuis	Weert
Máxima Medical Center	Veldhoven
Rode Kruis Hospital	Beverwijk
Dijklander Hospital	Purmerend
Zaans Medical Center	Zaandam

Eligible patients will be allocated randomly to either PDNS care or usual care in a 1:1 ratio, using a computer-generated list of random numbers. An independent researcher (who will not perform study assessments) will perform the randomization using an online data management system. Subsequently, this researcher will contact the PDNSs to inform them about which participants are randomized to the intervention group. The other participants will receive a letter or an e-mail stating that they have been assigned to the control group. To ascertain an equal representation of patients, we will stratify for gender and disease duration (according to predefined subgroups; i.e., disease duration < 5 years, 5–10 years, and > 10 years). The PDNS intervention will follow the Dutch guideline “Nursing care in Parkinson’s disease” [11] (see the “PDNS intervention” section below). A blinded researcher will perform the clinical assessments at baseline (t0), after 12 months (t1), and after 18 months (t2). Patients and caregivers will also be asked to complete a set of questionnaires at t0, t1, and t2. Finally, every 3 months, patients and their caregivers will complete a questionnaire about healthcare utilization, costs, and productivity loss. Care providers (e.g., neurologists) will not be blinded to the assigned interventions. We do not foresee any reason why unblinding of participants would be necessary.

### Inclusion and exclusion criteria

The inclusion and exclusion criteria for patients are kept purposefully broad in order to represent the full diversity of the PD spectrum and thus generate results that apply to real clinical practice. All patients with PD, regardless of disease severity or disease duration, male and female, aged 18 years or older at the time of PD diagnosis are eligible. We will only exclude patients for the following reasons:

- Lack sufficient knowledge of the Dutch language to complete questionnaires
- Have received care from a PDNS in the past 2 years
- Have a score < 18 on the Mini Mental State Examination [18] and < 12 on the Frontal Assessment Battery [19]
- Have a type of atypical parkinsonism caused by medication (e.g., neuroleptics), a metabolic disorder (e.g., Wilson’s disease), encephalitis, or a neurodegenerative disorder (e.g., multiple system atrophy, progressive supranuclear palsy, corticobasal syndrome)
- Residing in a nursing home or another type of residential care facility (because the PDNS is not operational there)
- Have any other medical or psychiatric disorder that, in the opinion of the researcher, may compromise participation in the study

### Recruitment

Patients will be approached within each hospital using one of three scenarios:

1. The involved neurologists in the hospitals identify eligible patients from their electronic patient file and inform these patients about the study in their clinic (when the patient is coming in for a consultation). A patient who agrees to be approached by a researcher will be provided with the patient information letter.
2. The neurologists identify eligible patients using their electronic patient file and subsequently approach them by directly sending out a letter including a short description of the study and a form on which patients can indicate if they want to receive any further information about the study or not. Only if patients actively indicate that they wish to be approached will the researcher contact them by telephone and send them the information letter.
3. The research team organizes an information meeting for patients in the participating center (where the PDNS and neurologist are also present). Here, the patient information letter will be handed out directly. Importantly, patients will be given sufficient time to consider their participation. If they are interested, they will be contacted by the research team at least 2 weeks after the information session.

### Training and coaching of Parkinson’s disease nurse specialists

Before the start of the study, we will organize a single training session with all participating PDNSs (one from each center). The goal of this meeting is to acquire commitment to the study and uniformity in workflow by reviewing the “Nursing care in Parkinson’s disease” guideline to explain the study specifics and to discuss practical issues related to the study intervention. In addition, PDNSs will be closely coached in order to optimize the intervention and adherence to the guideline. Every month, an experienced PD nurse from Radboudumc will have an individual intervention session with each PDNS, mainly to discuss difficult cases and to optimize the intervention and its uniformity. Finally, we will organize a video meeting every 3 months with all PDNSs to maintain their commitment, support each other, discuss difficulties related to the study, and give each other advice [20, 21].

Importantly, for the purpose of this study, we will implement an increase in nursing staff capacity for participating nurses. This will allow us to study the real impact of current usual care, which would not be achieved by adding a new set of specifically trained research nurses to the existing PDNS staff. The PDNSs are all graduated nurses (education level according to the European Qualifications Framework 6 or 7) with a certificate in Parkinson’s nursing. Furthermore, they have achieved a standard of competence as described in the “Nursing care in Parkinson’s disease” guideline [11].

### PDNS intervention

The PDNS intervention will be performed according to the Dutch “Nursing care in Parkinson’s disease” guideline published in 2015 [11]. The intervention is not standardized but tailored to the patients’ and caregivers’ needs. This includes the following:

- Asses*sment of individual care needs of people with PD and their caregivers*: At the start of the study, the PDNS performs a specific nursing assessment related to the medical, physical, psychological, and social domains.
- *Development of a patient-centered treatment plan that supports the patient and caregiver in self-management*: The PDNS composes a multidisciplinary plan based on the results of the individual assessment and as prioritized by the patient and caregiver (shared decision making). The treatment plan is developed according to the national self-management framework [22].
- *Specific nursing interventions*: The intervention varies across disease stages and is tailored to the specific problems and needs of individual patients and their caregivers. The “Nursing care in Parkinson’s disease” guideline for care describes general and specific nursing interventions. General interventions consist of providing information and education, disease management (e.g., considering advanced treatment options such as DBS), and monitoring (e.g., of caregiver burden). Specific nursing interventions are described for the following areas: mental function, fatigue, sleep, urogenital functions, sexuality, medication adherence, orthostatic hypotension, caregiver burden, coping, mobility, self-management, and dietary issues. (Table 2 provides examples of such interventions.)
- *Collaboration with other healthcare professionals.* The PDNS stimulates and supports multidisciplinary collaboration between healthcare professionals based on the individual patient-centered treatment plan. The PDNS also plays a pivotal role in timely referral to other healthcare professionals.

**Table 2:** Specific nursing interventions according to the Dutch guideline for nursing care in Parkinson’s disease that are also reminiscent of the Fundamentals of Care Framework

Area	Interventions
Mental function	Providing information and education Activation and supporting the creation of a day structure Supporting the caregiver
Fatigue	Supporting the intake of food with sufficient caloric value Promoting physical exercise Structuring daily activities
Sleep	Providing sleep hygiene advice (e.g., no alcohol or caffeine before sleep, no watching television or using the computer before sleep) Changing medication in consultation with the neurologist in case of nocturnal on/off fluctuations
Urogenital functions	Advising to drink 1.5–2.0 L of fluid per day Advising the reduction of fluid intake before sleep Advising the intake of food rich in fibers
Sexuality	Providing information and education Providing specific advice according to the type of sexual dysfunction (e.g., reduced sexual desire, erectile dysfunction)
Medication adherence	Providing information and education about the timing and intake of medication (e.g., with water, not with milk) Stimulating medication adherence
Orthostatic hypotension	Advising to wear support stockings Advising to have sufficient salt and fluid intake per day Providing advice about postural changes
Caregiver burden	Providing information and education Refer the caregiver for cognitive behavioral therapy Refer the caregiver to a Parkinson’s disease-specific support group
Coping	Advising mindfulness training Supporting patients and caregivers to view problems from different perspectives to develop new strategies for solving these problems Refer for cognitive behavioral therapy
Mobility	Applying cognitive movement strategies Applying external cues Stimulating the patient to perform sufficient physical exercise
Self-management	Stimulating the patient to ask questions Providing individualized patient-related information Asking if the provided information matches the patient’s question
Dietary issues	Providing information and education about problems with food absorption (e.g., because of the interaction with protein intake) Preventing accidental weight loss Providing advice about oral care

Dutch guideline for nursing care in Parkinson’s disease [11]; Fundamentals of Care Framework [23]. Note that this list is not exhaustive

The PDNS will maintain a predefined electronic study report according to a structured format for each patient with PD, documenting the individual care needs, current symptoms, performed interventions, and (changes in) the individual care plan. This report will be started at the initial assessment and updated at every follow-up contact with the patient, such as at the outpatient clinic, during a telephone consultation, or at a home visit. This information will be purposefully collected for a possible process analysis at the end of the study.

Patients will have regular contact with their PDNS about the progress and realization of the personal goals, both during face-to-face contacts and by telephone, and sometimes during additional home visits. The frequency and type of contact will be optimized for each patient, depending on disease stage and individual patient needs. The “Nursing care in Parkinson’s disease” guideline advises that each patient have a minimum of one contact with the PDNS each year [11]. Currently in the Netherlands, patients are seen, on average, twice annually by their PDNS, with an additional two interim telephone consultations per year.

The control group will receive ongoing usual care that is medically comparable to that in the intervention group, but without a nursing intervention. This involves regular consultations with a neurologist in their own community hospital (typically two to four times per year, depending on patient preferences and health status). In addition, control patients will have no restrictions when considering any other medical treatments (e.g., by a psychologist or social worker). Importantly, many key elements of care (including in particular the treating neurologist) remain comparable between the two arms because of the randomization at the patient level within hospitals.

### Clinical assessment and outcome measures

At baseline, t1, and t2, all patients will visit their own hospital for the study assessments, which are performed by a blinded researcher (Parkinson’s Disease Questionnaire [PDQ-39], Movement Disorders Society-sponsored revision of the Unified Parkinson’s Disease Rating Scale [MDS-UPDRS], and Timed Up and Go test [TUG]). The patients and their caregivers will also complete home questionnaires. In addition, every 3 months, patients will receive a questionnaire at home regarding healthcare utilization, costs, and productivity loss over the past 3 months. Caregivers will complete a cost questionnaire, including healthcare utilization, costs, and productivity loss, specifically related to caregiver burden. To improve adherence, patients and caregivers can choose whether they prefer to fill out digital or paper-based questionnaires. Participants will be contacted by telephone when they do not complete the questionnaires within 4 weeks. All the outcomes, including secondary outcome measures, can be found in Table 3.

**Table 3:** Outcome measures used at different time points

Outcome	Questionnaire	Baseline	12-month follow-up	18-month follow-up	Every 3 months
Co-primary outcome measures					
Quality of life	Parkinson’s Disease Questionnaire (PDQ-39) [24]	X	X	X	
Motor symptoms	Movement Disorders Society-sponsored revision of the Unified Parkinson’s Disease Rating Scale part III (MDS-UPDRS part III) [25]	X	X	X	
Secondary outcome measures (patient-related)					
Longitudinal PD symptoms	Movement Disorders Society-sponsored revision of the Unified Parkinson’s Disease Rating Scale parts I, II, and IV (MDS-UPDRS parts I, II, and IV) [25]	X	X	X	
Mobility	Timed Up and Go (TUG) [26]	X	X	X	
Nonmotor symptoms (anxiety and depression)	Hamilton Anxiety and Depression Scale (HADS) [27]	X	X	X	
Nonmotor symptoms (e.g., sleep, incontinence, constipation)	Scales for Outcomes in Parkinson’s Disease–Autonomic Questionnaire and Sleep Questionnaire (SCOPA-AUT) [28], SCOPA-SLEEP [29]	X	X	X	
Health-related quality of life	EuroQoL-5D (EQ-5D) [30]	X	X	X	
Experienced quality of care	Consumer Quality Index (CQI) [31]	X	X	X	
Self-management	Patient Activation Measure (PAM13) [32]	X	X	X	
Medication adherence	Morisky Medication Adherence Scale (MMAS) [33]	X	X	X	
Secondary outcome measures (caregiver-related)					
Health-related quality of life	EuroQoL-5D (EQ-5D) [30]	X	X	X	
Caregiver burden [24]	Zarit Caregiver Burden Index (ZBI) [34]	X	X	X	
Caregiver quality of life	CarerQol-7D [35]	X	X	X	
Skills of proactive coping	Utrecht Proactive Coping Competence Scale (UPCC) [36]	X	X	X	
Healthcare utilization, costs, and productivity loss					
Medical consumption of the patient	Medical Consumption Questionnaire (MCQ) [37]				X
Productivity loss of the patient	Productivity Cost Questionnaire (PCQ) [38]				X
Medical consumption of the caregiver related to caregiver burden	Medical Consumption Questionnaire (MCQ) specifically adapted for andaimed at caregivers [37]				
Productivity loss of the caregiver related to caregiver burden	Productivity Cost Questionnaire (PCQ) specifically adapted for and aimed at caregivers [38]				X

*PD* Parkinson’s disease

Similar to previous large randomized controlled trials in the field of PD [39, 40], we will use two co-primary outcomes: quality of life and motor symptoms [41]. For measuring quality of life, we will use the PDQ-39, which is the most widely used quality-of-life scale in PD and frequently used as an outcome measure, such as in trials on DBS [42] and multidisciplinary care [43]. Our second co-primary outcome measure is the severity of motor symptoms measured by the MDS-UPDRS part III. The MDS-UPDRS is a frequently used clinical rating scale and has been shown to be sensitive to change in clinical status [44]. Both scales have been validated previously and are reliable and valid methods to measure either quality of life [45] or motor symptoms [46] in people with PD.

### Data collection and management

Patients will be given a unique personal identification code not containing any information that refers back to the individual. The key file connecting personal identification codes to the individual patient will be stored on a secure Radboudumc data server. Only the research team has access to this key. The key file will be stored on a different server from the one with acquired study data for 5 years, allowing the research team to contact patients after they have finished the study. After 5 years, the key file will be destroyed.

Data from all paper-based case report forms (CRFs) completed by the researcher (PDQ-39, MDS-UPDRS, and TUG) will be entered manually into an online certified data management system (Castor EDC; Castor, Amsterdam, the Netherlands). Online CRFs (the remaining questionnaires) will automatically be recorded in Castor EDC. When patients or caregivers are not able to complete questionnaires online, they also have the opportunity to do this on paper. We will send out the questionnaires by post, and patients can return the completed questionnaires using a self-addressed envelope. These questionnaires will be entered manually into Castor EDC. Both online and paper-based CRFs only contain the personal identification code.

Clinical notes taken by the PDNS in the online study report will also not contain any information that refers back to the individual. PDNSs are instructed to make notes according to a predefined structured format without mentioning personal information that traces back to an individual patient. The study report will be completed in Castor EDC.

### Adverse events

All serious adverse events (SAEs) will be collected and followed up by the investigators and documented in the electronic CRFs. Each SAE will be reported by the respective PDNS to the study team (DR), and the SAE will be reported to the local ethics committee as soon as the researcher has knowledge of the SAE, but no later than 24 h after the researcher has become aware of the event. Other adverse events will not actively be inquired for during the study, because of the low risk associated with the trial. When a participant spontaneously reports an adverse event, it will be registered in the electronic CRF.

### Sample size analysis

We performed a sample size calculation based on the PDQ-39 score. On the basis of observations in one of our previous studies in a similar population of patients with PD where we evaluated multidisciplinary care [43], we found a mean improvement in PDQ-39 score in the intervention group of − 2.5 (SD, 5.8) points and a mean deterioration in PDQ-39 score in the control group of + 1.4 (SD, 8.6). We calculated the sample size based on a mean difference between groups of 3.9, with an SD of 8.6 (the highest SD reported). Using a significance level of alpha = 0.025 (instead of 0.05 because of two primary endpoints: PDQ-39 and MDS-UPDRS part III) and a power of 80%, a sample of 93 patients in each group would be needed. Considering an attrition rate of 20%, 117 patients are needed per group. We have rounded this up to 120 patients per group, which means a total of 240 patients. We expect this to be feasible because following a baseline inventory, all centers indicated that they would be able to include at least 30 patients.

### Data analysis

The economic evaluation investigates, alongside the clinical trial, the value for money of full implementation of the PDNS into PD care from a societal and healthcare perspective. We will take all relevant costs into account. The cost-effectiveness time frame adheres to the clinical study protocol and evaluates cost-effectiveness up to 18 months after randomization. Cost will be measured using a healthcare utilization questionnaire (e.g., including medical consultations, hospital admissions, medication, travel costs) and a questionnaire measuring productivity loss while working of both patients and caregivers. Per item of healthcare consumption, standard cost prices will be determined using the guideline for performing economic evaluations [47]. If standardized prices are not available, full cost prices will be determined using activity-based costing. Costs will be analyzed using a mixed model approach or a general linear model approach with a gamma distribution using a log link to account for possible skewness of the cost data.

We will use a PD-specific quality of life measure (PDQ-39) and a generic health-related quality of life scale (EQ-5D) to evaluate the quality of the health status of patients. The potential difference in quality-adjusted life-years measured with the EQ-5D will be analyzed with a regression approach. We will use a linear mixed model with repeated measurements to test for differences in quality of life (measured with the PDQ-39) between both groups. The same analysis will be used to measure differences between groups in the secondary outcome measures. We will include study center as a random effect and fixed effects for group, time, and the interaction between group and time. Each of the outcomes will be included as a dependent variable. Statistical analyses will be performed on the basis of the intention-to-treat principle.

As mentioned previously, we hypothesize that both interventions (PDNS care versus no PDNS care) will yield equal costs, while PDNS care is more effective. If this hypothesis is confirmed, then the effect analysis is sufficient to show the efficiency of PDNS care. The design of the economic evaluation follows the principles of a cost-effectiveness analysis and adheres to the Dutch guideline for performing economic evaluations in healthcare [47].

Besides the overall cost-effectiveness evaluation, we will perform a preplanned subgroup analysis based on disease duration (diagnosis made < 5 years, 5–10 years, or > 10 years ago) to obtain more insight into the nursing interventions used in each disease stage and the effects of PDNS care in these different groups of patients. This subgroup analysis will be performed because, for example, for the more severely affected patients, the nursing intervention is expected to become more intensive and possibly more effective but also more expensive. When different patterns of this kind are found, this should be investigated further in future trials that are powered adequately to address such group differences.

### Trial oversight

The chief investigator has the overall responsibility for the conduct of the study. The study group has responsibility for the day-to-day management of the trial and consists of the following authors: DLMR, HHL, RHH, MM, NMdV, and BRB, who designed the study. DLMR and NMdV are responsible for day-to-day management of the trial, including the inclusion of participants and communication with participating centers, participants, and the ethics committee. TvA, CCSD, and HV have a more consultative role and provide substantial feedback regarding the trial procedures. There will be no independent data monitoring committee, owing to the low risk associated with the trial. The results of the study will be sent for publication to a peer-reviewed medical journal. No professional writers will be involved. In addition, the results will be shared with trial participants via the Dutch Parkinson Association and via ParkinsonNet. We report no restrictions for publication.

## Discussion

Here, we present the rationale and design of the NICE-PD study, a large (*n* = 240) randomized controlled clinical trial that aims to evaluate the cost-effectiveness of specialized nursing interventions provided by a PDNS for people with PD. The results of this trial will have an immediate impact on current care for people with PD, independent of its outcome. When the intervention is shown to be cost-effective, a wider implementation of PDNS care for all patients will be warranted. This requires an increase in PD nursing capacity, which means that further efforts must then be initiated to ensure that policy makers and payers will invest in the reimbursement of PDNS care. We expect that investment in extra PDNS capacity will not lead to a net increase in costs, because the number of neurologist consultations may decrease proportionally to the increasing PDNS care. On the other hand, if cost-effectiveness is not shown, current guideline recommendations should be reevaluated critically, and a discussion should be started on how PDNS care delivery should be modified for it to be more effective. One may also argue that PDNS care could be considered successful when quality of life significantly improves with a slight increase in costs. With this outcome, it may be worth investing in PDNS care to further improve the quality of life of people with PD and to search for solutions to optimize efficiency and reduce costs of PDNS care interventions.

We hypothesize that offering PDNS care will lead to higher quality of life with equal healthcare costs. Increasing direct medical costs (for nurse staffing) are expected to be offset by a reduced number of (telephone) consultations with the general practitioner and neurologist. These short-term goals are the focus of the present NICE-PD proposal. In addition to the short-term effects, we also expect long-term benefits, but these are beyond the scope of the current project. Examples of potential long-term benefits include a reduction in the number of nursing home admissions and fewer emergency visits to the hospital, which could potentially lead to a substantial cost reduction.

This is the first randomized controlled clinical trial to evaluate the cost-effectiveness of PDNS care. However, this study is not without challenges. During the study period, each hospital receives extra budget (out of the grant money) to increase their PDNS capacity for providing better care to the patients in the intervention group. However, this reimbursement cannot be continued after the study has ended. It will be a challenge to offer continuity of care for the participating patients when nursing capacity has to be reduced again after the study because of a reduction in funding. We hope that positive results of the present study will provide a strong impetus for identifying the necessary financial resources to finance sufficient nursing capacity in the long term. To ascertain this, we will engage in discussions with Dutch payers already at the outset of the study. The second challenge is that the patients in the control group will not have access to PDNS care during the complete study period. However, there are no restrictions on other medical treatments, which means that the control patients are allowed to consult all other available healthcare providers (e.g., physiotherapists, psychologists, and social workers). There are two exceptions, though, in which care by a PDNS will only be directed at these specific situations: (1) providing specific information and guidance about advanced therapies (e.g., DBS, duodopa infusion) and (2) moral dilemmas in crises (e.g., psychosis). Note that a group of patient researchers who were involved in the design of this NICE-PD study consider it ethically acceptable to deny these patients access to PDNS care during a period of 18 months because we are not withholding an evidence-based treatment (current evidence is limited). This is because patients were not receiving any PDNS care anyway outside the study and because we can now exploit this situation to gain scientific evidence about the cost-effectiveness of PDNS care. This new knowledge will eventually benefit all patients with PD, including those allocated to the control arm of the present trial.

Third, because PDNSs are not operational in nursing homes or other types of residential care facility, it may be more difficult to include severely affected patients in this study. We will try to overcome this by stratifying for disease duration and by carefully selecting patients in all disease stages from each center. Finally, because quality of life is a very generic outcome measure, it may be a challenge to find relevant results on this metric. To overcome this challenge, we have chosen to use two co-primary outcome measures. To accommodate this, we have chosen more cautious levels of statistical significance. Moreover, the study was powered for a single outcome (quality of life), but the study’s power is sufficient to also detect a minimal clinically important difference for the co-primary outcome (the MDS-UPDRS). The reason why we expect an improvement in this other primary outcome is because when patients receive more integrated care, their motor symptoms may also improve.

In conclusion, this study will generate new insights into the cost-effectiveness of specialized PD nursing interventions for people with PD. If positive results are found, a large shift in the organization of PD care is needed to warrant equal access to PDNS care for every person with PD.

### Trial status

Protocol version 3, date: April 8, 2019. Recruitment started on January 7, 2019, and is currently ongoing. The expected date for recruitment completion is December 2019.

## Supplementary information

**Additional file 1.** The NICE-PD SPIRIT 2013 checklist.

## Data Availability

On the consent form, participants will be asked whether they agree with the following statement: “I know that participating in this trial is voluntary. I also know that at any time I can decide to withdraw from the trial. I do not have to give any reason. The data that is collected until that moment, will be used for the research. A number of people are allowed to view my data. These include the members of the research team, the Ethical Committee, people that verify the safety of the trial (monitor), and the Dutch Healthcare Inspection.” This trial does not involve collecting biological specimens for storage. The aggregated datasets analyzed during the current study will be available from the corresponding author upon reasonable request.

## Abbreviations

CONSORT: Consolidated Standards of Reporting Trials
CRF: Case report form
DBS: Deep brain stimulation
MDS-UPDRS: Movement Disorders Society-sponsored revision of the Unified Parkinson’s Disease Rating Scale
NICE-PD: Cost-effectiveness of Nursing Interventions for Patients With PDPD Parkinson’s disease
PDNS: Parkinson’s disease nurse specialist
PDQ-39: Parkinson’s Disease Questionnaire
SAE: Serious adverse event
SPIRIT: Standard Protocol Items*:* Recommendations for Interventional Trials
TUG: Timed Up and Go
